# Biosmart Materials: Breaking New Ground in Dentistry

**DOI:** 10.1155/2014/986912

**Published:** 2014-02-02

**Authors:** Vijetha Badami, Bharat Ahuja

**Affiliations:** Department of Conservative Dentistry and Endodontics, A.M.E'S Dental College, Hospital and Research centre, Bijengere Road, Raichur 584103, India

## Abstract

By definition and general agreement, smart materials are materials that have properties which may be altered in a controlled fashion by stimuli, such as stress, temperature, moisture, pH, and electric or magnetic fields. There are numerous types of smart materials, some of which are already common. Examples include piezoelectric materials, which produce a voltage when stress is applied or vice versa, shape memory alloys or shape memory polymers which are thermoresponsive, and pH sensitive polymers which swell or shrink as a response to change in pH. Thus, smart materials respond to stimuli by altering one or more of their properties. Smart behaviour occurs when a material can sense some stimulus from its environment and react to it in a useful, reliable, reproducible, and usually reversible manner. These properties have a beneficial application in various fields including dentistry. Shape memory alloys, zirconia, and smartseal are examples of materials exhibiting a smart behavior in dentistry. There is a strong trend in material science to develop and apply these intelligent materials. These materials would potentially allow new and groundbreaking dental therapies with a significantly enhanced clinical outcome of treatments.

## 1. Introduction

Materials science is not what it used to be. Traditionally materials used in dentistry were designed to be passive and inert, that is, to exhibit little or no interaction with body tissues and fluids. Materials used in the oral cavity were often judged on their ability to survive without interacting with the oral environment.

The present scenario has changed. Many of the advanced materials at the forefront of materials science are functional: they are required to perform things and to undergo purposeful change. They play an active part in the way the structure or device works.

Perhaps the first inclination that an “active” rather than “passive” material could be attractive in dentistry was the realisation of the benefit of fluoride release from materials. This both reflects and permits a change in material philosophy. The same is true in many other areas of engineering, such as aerospace, automotive engineering, biomedicine, and robotics.

Materials used in dentistry can be classified as bioinert (passive), bioactive, and bioresponsive or smart materials based on their interactions with the environment.

Smart materials can be defined as designed materials that have one or more properties that can be significantly changed in a controlled fashion by external stimuli, such as stress, temperature, moisture, pH, and electric or magnetic fields [1]. These materials are also referred to as responsive materials.

Smart materials have been around for many years and they have found a large number of applications. The use of the terms “smart” and “intelligent” to describe materials and systems came from the USA and started in the 1980s despite the fact that some of these so-called smart materials had been around for decades. Early smart material applications started with magnetostrictive technologies. This involved the use of nickel as a sonar source during World War I to find German U-boats by Allied forces.

Smart behaviour occurs when a material can sense some stimulus from its environment and react to it in a useful, reliable, reproducible, and usually reversible manner. A really smart material will use its reaction to the external stimulus to initiate or actuate an active response. Smart materials can happen by chance or they can be designed to incorporate smartness in them.

Some researchers insist that no material by itself is truly smart, as opposed to being simply responsive. They insist that being smart is not just a matter of producing a response in proportion to a stimulus, but includes principles such as adaptation and feedback. Others draw a distinction between merely smart and truly intelligent, in the sense of being able to do things like make decisions or repair oneself. No artificial materials are yet intelligent in this sense.

This paper aims to describe the various materials in dentistry that exhibit some sort of smart behaviour.

## 2. Nickel-Titanium Smart Alloy

The term “smart material” or “smart behaviour” in the field of dentistry was probably first used in connection with Nickel-Titanium (NiTi) alloys, or shape memory alloys (SMAs), which are used as orthodontic wires. The shape memory effect was first observed in copper-zinc and copper-tin alloys by Greniger and Mooradian in 1938. Nickel-Titanium was developed 50 years ago by Buehler et al. in the Naval Ordinance Laboratory (NOL) in Silver Springs, Maryland. In endodontics, 55 wt% Ni and 45 wt% Ti are commonly used, referred to as “55NiTiNOL.” NiTi was introduced to endodontics by Walia et al. in 1988.

The smart behavior of NiTi alloys is because of two salient features called “superelasticity” and “shape memory.” This “smart” property is the result of the substance's ability to undergo a phase change—a kind of atomic ballet in which atoms in the solid subtly shift their positions in response to a stimulus like a change in temperature or application of mechanical stress.

**Figure 1:**
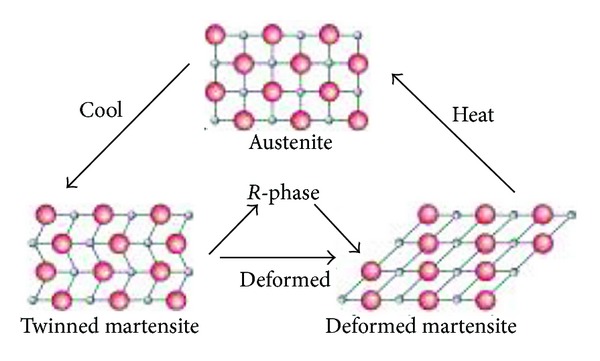
Force and temperature dependent transitions from austenite to martensite, including the intermediary *R*-phase.

**Figure 2:**
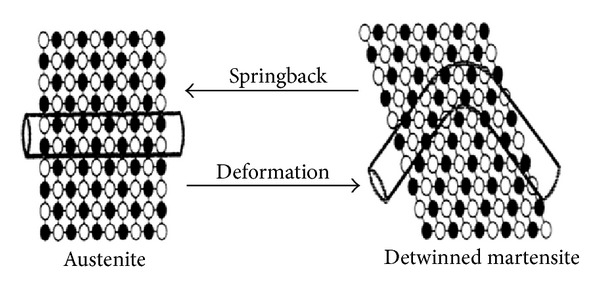
Diagrammatic representation of the superelasticity effect of NiTi alloy. Source: [33].

**Figure 3:**
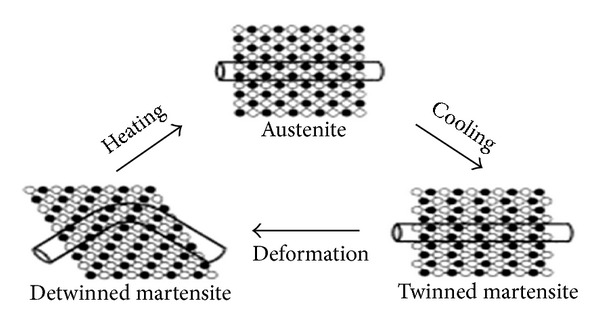
Diagrammatic representation of the shape memory effect of NiTi alloy. Source: [33].

Nitinol basically exists in two phases. The low temperature phase is called the martensitic or daughter phase (a body centered cubic lattice) and the high temperature phase is called the austenitic or parent phase (hexagonal lattice). This lattice organisation can be altered either by stress or temperature (Figure 1). In endodontics, the root canal treatment causes stress to NiTi files and a stress-induced martensitic transformation occurs from the austenitic to the martensitic phase within the speed of the sound. A change in shape occurs, together with volume and density changes. This ability of resisting stress without permanent deformation, going back to initial lattice form, is called superelasticity (Figure 2). Shape memory effect is the ability of the NiTi file to come back to its original straight form without showing any sign of lasting deformation [2]. When an SMA is cold, or below its transformation temperature, it has a very low yield strength and can be deformed quite easily into any new shape, which it will retain. However, when the material is heated above its transformation temperature, it undergoes a change in crystal structure which causes it to return to its original shape (Figure 3).

The superelasticity of NiTi rotary instruments provides improved access to curved root canals during the chemicomechanical preparation, with less lateral force exerted. It allows more centered canal preparations with less canal transportation and a decreased incidence of canal aberrations. Nitinol normally exists in an austenitic crystalline phase that transforms to a martensitic structure on stressing at a constant temperature. In this martensitic phase, only a light force is required for bending. If the stress is released, the structure recovers to an austenitic phase and its original shape. This phenomenon is called stress-induced thermoelastic transformation [3].

The superelasticity of NiTi allows deformations of as much as 8% strain to be recoverable in comparison with a maximum of less than 1% with stainless steel. This is critical for rotary endodontic instruments for two reasons. During preparation of curved canals, forces between the canal wall and abrading instruments are smaller with more elastic instruments; hence, fewer preparation errors will likely occur. Second, rotation in curved canals will bend instruments once per rotation, which will ultimately lead to work hardening and brittle fracture, also known as cyclic fatigue [4].

External stresses transform the austenitic crystalline form of NiTi into a martensitic crystalline structure that can accommodate greater stress without increasing the strain. When the material is in its martensite form, it is soft and ductile and can easily be deformed. SE NiTi is highly elastic, whereas austenitic NiTi is quite strong and hard [5].

In orthodontics, NiTi arch wires are used instead of stainless steel owing to their limited flexibility and tensile properties. NiTi wires, because of their superelasticity and shape memory, apply continuous gentle forces on the teeth, which are in physiologic range over a longer period of time [6].

## 3. Smart Composites

Generally, Boskey mentioned that Aaron S. Posner to have firstly described amorphous calcium phosphate (ACP) [7] in the mid-1960s. It was obtained as an amorphous precipitate by accident when mixing high concentrations (30 mM) of calcium chloride and sodium acid phosphate (20 mM) in buffer. ACP based materials have been developed for a number of applications like bases/liners, orthodontic adhesives, endodontic sealers [8], and as pit and fissure sealants.

ACP has been evaluated as a filler phase in bioactive polymeric composites. Skrtic has developed unique biologically active restorative materials containing ACP as filler encapsulated in a polymer binder, which may stimulate the repair of tooth structure because of releasing significant amounts of calcium and phosphate ions in a sustained manner. In addition to excellent biocompatibility, the ACP containing composites release calcium and phosphate ions into saliva milieus, especially in the oral environment caused by bacterial plaque or acidic foods. Then these ions can be deposited into tooth structures as apatitic mineral, which is similar to the hydroxyapatite (HAP) found naturally in teeth and bone [9]. ACP at neutral or high pH remains as ACP. When low pH values (at or below 5.8) occur during a carious attack, ACP converts into HAP and precipitates, thus replacing the HAP lost to the acid. So, when the pH level in the mouth drops below 5.8, these ions merge within seconds to form a gel. In less than 2 minutes, the gel becomes amorphous crystals, resulting in calcium and phosphate ions [10]. This response of ACP containing composites to pH can be described as smart.

## 4. Self-Healing Composites

Materials usually have a limited lifetime and degrade due to different physical, chemical, and/or biological stimuli. These may include external static (creep) or dynamic (fatigue) forces, internal stress states, corrosion, dissolution, erosion, or biodegradation. This gradually leads to a deterioration of the materials structure and finally failure of the material [11].

Nature has inspired scientists and researchers to develop materials which can repair by themselves. A great many natural *“materials”* are themselves self-healing composite materials. An example for this is natural bone which is permanently remodelled and which can self-repair (heal) even after a major fracture has occurred. A key focus of current scientific research is the development of bioinspired materials systems [12].

**Figure 4:**
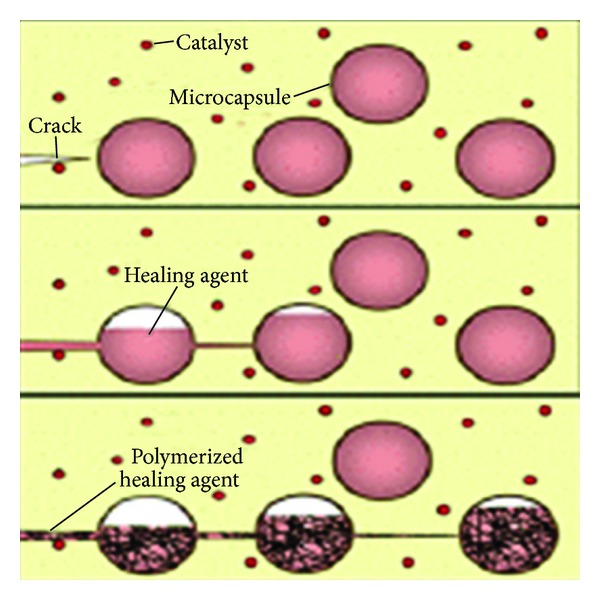
Basic method of the microcapsule approach (White et al., 2001) Source: [12].

One of the first self-repairing or self-healing synthetic materials reported interestingly shows some similarities to resin-based dental materials, since it is resin-based. This was an epoxy system which contained resin filled microcapsules. If a crack occurs in the epoxy composite material, some of the microcapsules are destroyed near the crack and release the resin. The resin subsequently fills the crack and reacts with a Grubbs catalyst dispersed in the epoxy composite, resulting in a polymerization of the resin and a repair of the crack (Figure 4). Similar systems were demonstrated to have a significantly longer duty cycle under mechanical stress in situ compared to similar systems with the self-repair [11].

It can be expected that dental composites using this technology would have a significantly longer duty cycle and enhanced clinical performance. Problems may arise from the potential toxicity of the resins in the microcapsules and from the catalyst, which needs to be present in the composite. The amounts of these agents necessary to repair microcracks in the dental composite, however, seem to be rather small, and may well be below the toxicity threshold [11].

The self-repairing mechanism based on microcapsules may be more promising, and composites repaired in that way may perform better than those repaired with macroscopic repair approaches, some of which [13] have been shown not to lead to satisfactory mechanical properties of the repaired composite with an aesthetically pleasing result. These conventional composites, however, have little in common with natural tooth structure and do not support any kind of tissue regeneration.

Wertzberger et al. [14] conducted a study to determine the efficacy of self-healing of a highly filled composite and to investigate the physical properties of a model dental compound formulated to automatically heal cracks. A visible light cured model resin consisting of Triethylene Glycol Dimethacrylate (TEGMA) : Urethane Dimethacrylate (UDMA) : Bisphenol A Glycidyl Methacrylate (BisGMA) (1 : 1 : 1) at 45% w/w with silane 0.7 *μ* glass was formulated with a self-healing system consisting of encapsulated dicyclopentadiene and Grubbs catalyst. The base resin was also formulated and characterized with the microcapsules alone, Grubbs catalyst alone, and no healing additives. Fracture toughness (KIc) was assessed using single edge notch specimens in three-point bend. The fracture toughness of the self-healing material was statistically similar to the control. The modulus decreased in the composites with encapsulated dicyclopentadiene.

## 5. Smart Ceramics

Aesthetics is one of the major concerns in dentistry. Ceramics, though available since a long time to fabricate crowns, have been used with a metal substructure as porcelain fused metal (PFM) crowns. This metal substructure reduces the aesthetic quality of the restoration. A high tech ceramic zirconia is now available that has already been proven in many extreme situations such as heat shields in space shuttle, brake discs of sports cars, and spherical heads of artificial hip joints.

Zirconia are polycrystalline ceramics that do not contain glass. All of the atoms are packed into regular crystalline arrays through which it is much more difficult to drive a crack than it is through atoms in the less dense and irregular network found in glasses. Hence, polycrystalline ceramics generally are much tougher and stronger than glass-based ceramics. Well-fitting prostheses made from polycrystalline ceramics were not practical before the availability of computer-aided manufacturing. In 1995, the first “all ceramic teeth bridge” was fabricated at ETH Zurich based on a process that enabled direct machining of bridges.

In general, these computer-aided systems use a 3D data set representing either the prepared tooth or a wax model of the desired substructure. The systems use this 3D data set to create an enlarged die upon which ceramic powder (Procera, Nobel Biocare, Göteborg, Sweden) is packed or to machine an oversized part for firing by machining blocks of partially fired ceramic powder (ZirCAD, Ivoclar Vivadent; Cercon Zirconia, Dentsply Prosthetics, York, Pa.; Lava Zirconia, 3 M ESPE, St. Paul, Minn.; Vita In-Ceram YZ, Vita Zahnfabrik). Both of these approaches rely on well-characterized ceramic powders (i.e., tight control over particle sizes and packing density) for which firing shrinkages can be predicted accurately [15].

The fracture toughness and flexural strength of zirconia are significantly higher than that of alumina or any other currently available ceramic. Although most dental zirconia is a bit opaque and copings need to be veneered for high aesthetics, these prostheses can be quite lifelike. The fracture toughness of the most interesting polycrystalline ceramic now available for dentistry, transformation toughened zirconia, involves an additional mechanism not found in other polycrystalline ceramics. Unlike alumina, zirconium oxide is transformed from one crystalline state to another during firing. At firing temperature, zirconia is tetragonal and at room temperature monoclinic, with a unit cell of monoclinic occupying about 4.4% more volume than when tetragonal. Unchecked, this transformation was a bit unfortunate since it would lead to crumbling of the material on cooling. In the late 1980s, ceramic engineers learned to stabilize the tetragonal form at room temperature by adding small amounts (app. 3–8 mass%) of calcium and later yttrium or cerium [16]. Although stabilized at room temperature, the tetragonal form is really only “metastable,” meaning that trapped energy still exists within the material to drive it back to the monoclinic state. It turned out that the highly localized stress ahead of a propagating crack is sufficient to trigger grains of ceramic to transform in the vicinity of that crack tip. In this case, the 4.4% volume increase becomes beneficial, essentially altering material conditions around the crack tip, shielding it from the outside world (more formally stated, transformation decreases the local stress intensity) [17].

**Figure 5:**
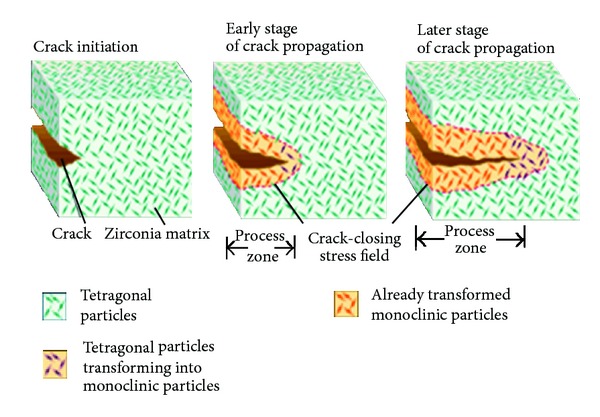
Phase transformation in Zirconia. Source: Internet.

The result is that a compressive or crack closure stress is produced which slows down or stops the crack. This crystallographic transformation (Figure 5) in response to stress makes zirconia a smart material.

## 6. Glass Ionomer Cement as a Smart Material

Wide temperature fluctuations may occur in the oral cavity due to the intake of hot or cold food and fluids. Hence, the restorative materials placed in this environment may show thermal expansion or contraction in response to thermal stimuli. The coefficient of thermal expansion (CTE) is normally used to describe the dimensional changes of a substance in response to thermal change [18].

The CTE is an inherent characteristic of each material at a specific temperature. When dealing with thermally induced volumetric changes, comparison of CTE values of the restorative material and the tooth substance is more important than the CTE value of the material itself [19]. When two materials expand or contract at a similar rate, gap formation at the interface is almost a nonissue; thus, microleakage is negligible [20, 21].

The mismatch of thermal expansion and contraction between a restoration and the tooth structure may cause stresses to develop at the interface and this may have unfavourable effects on the margins and finally lead to microleakage [18].

The vast majority of materials respond to a temperature change in a predictable manner. When samples of restorative materials were heated in order to determine their values of coefficient of thermal expansion, an interesting observation was made. For composite materials, expansion and contraction occurred in the expected way and a coefficient could readily be determined, and whether testing was done dry or wet made little or no difference [22]. For glass-ionomers, little or no change in dimension was observed when heating and cooling between 20°C and 50°C in wet conditions [18]. In dry conditions, the materials showed a marked contraction when heated above 50°C. The explanation for this behaviour is that the expected expansion on heating is compensated by fluid flow to the surface of the material to cause a balancing of the dimensional changes. On cooling, the process was reversed. In dry conditions, the rapid loss of water on heating results in the observed contraction. This behaviour is akin to that of human dentine where very little dimensional change is observed on heating in wet conditions and a marked contraction is noted in dry conditions. Both results can be explained by flow of fluids in the dentinal tubules. Hence, the glass-ionomer materials can be said to be mimicking the behaviour of human dentine through a type of smart behaviour [23].

The mass loss of glass-ionomer cements (GICs) in wet conditions is significantly less than that in dry conditions (*P* < 0.05). GICs, as water based materials, have the ability to exchange fluid with the environment. The “loosely” bound water is readily lost and regained as a result of changes in the environmental conditions (e.g., temperature). The loss of “loosely bound” water is likely to be a reversible process; that is, the water may be reabsorbed on cooling. The water gained from the environment may compensate for the contraction of the GIC matrix. Therefore, the final dimensional change of GICs in wet ambient conditions may be minimal as a net result of thermal expansion and contraction, water loss, and water gain. Hence, the reaction of GICs to their environment is active and they may be considered as having “smart” behaviour. The initial water loss caused by environmental temperature change may be considered as the “trigger” to this “smart” behaviour [18].

Porosity is an inherent property of GICs [24, 25] and the fluid contained in the porosities may contribute significantly to the water content.

Both the method of mixing and the viscosity of the cement have an effect on porosity. In the low viscosity material, hand mixing reduces the porosity significantly compared to mechanical mixing, either by shaking or rotation. For the viscous material, the levels of porosity are low and not significantly affected by mixing. These differences in porosity are reflected in differences in water absorption. Hence, this aspect of the smart behaviour of dental cements can be controlled by the operator [23].

GICs are described as “smart materials” with respect to their thermal behaviour, since it is a desired feature, when restorative materials undergo thermally induced volumetric changes close to those of the tooth substance [18, 19].

## 7. Smartseal Obturation System

Obturation of root canals should prevent reinfection of the canal space and ultimately prevent periradicular disease. This objective may be achieved by three-dimensional filling of the instrumented canal, accessory canals, and dead spaces. While different canal filling techniques are currently available to achieve this goal, there is ongoing interest in developing simplified obturating materials/techniques for filling irregular shaped canals and to minimise voids created during obturation procedures, which may act as nidi for growth of residual biofilms.

The C Point system (EndoTechnologies, LLC, Shrewsbury, MA, USA) is a point-and-paste root canal filling technique that consists of premade, hydrophilic endodontic points and an accompanying sealer. The deformable endodontic point (C Point) is available in different tip sizes and tapers and is designed to expand laterally without expanding axially, by absorbing residual water from the instrumented canal space and that from naturally-occurring intraradicular moisture [26]. The inner core of C Point is a mix of two proprietary nylon polymers: Trogamid T and Trogamid CX. The polymer coating is a cross-linked copolymer of acrylonitrile and vinylpyrroli done, which has been polymerised and cross-linked using allyl methacrylate and a thermal initiator. The lateral expansion of C Point is claimed to occur nonuniformly, with the expandability depending on the extent to which the hydrophilic polymer is prestressed (i.e., contact with a canal wall will reduce the rate or extent of polymer expansion) [27]. This nonisotropic lateral expansion is said to enhance the sealing ability of the root canal filling, thereby reducing the possibility of reinfection and potentiating the long-term success of root canal treatment. As claimed by the manufacturer, although C Point is capable of achieving a relative good fit of an irregular canal space, gaps may still remain between the walls of the canal and the expanded point. Consequently, an accompanying sealer must be used to seal those areas [28].

Smartpaste bio is a resin-based sealant designed to swell through the addition of ground polymer. The manufacturer claims that the addition of bioceramics gives the sealer exceptional dimensional stability and makes it nonresorbable inside the root canal. Smartpaste bio produces calcium hydroxide and hydroxyapatite as by-products of the setting reaction, rendering the material both antibacterial while setting and very biocompatible once set. Also, it has a delayed setting time (4–10 hr) and is hydrophilic in nature, allowing the propoint to hydrate and swell to fill any voids.

Economides et al. (2011) evaluated, ex vivo, the push-out bond strength of Smartseal compared with gutta-percha/AH-26. The study revealed no significant differences (*P* > 0.05) between the mean bond strengths of the various groups, thus indicating that there was no difference in adhesion to dentine between the Smartseal system and gutta-percha/AH-26 applied using either the single cone or lateral condensation technique [29].

Eid et al. (2013) evaluated the biocompatibility of C Point and commercially available gutta-percha points using a rat odontoblast-like cell line (MDPC-23) by measuring cell viability and mineralization potential of MDPC-23 cells. They concluded that the in vitro biocompatibility of C Point is comparable to gutta-percha with minimal adverse effects on osteogenesis after elution of potentially toxic components [30].

Didato et al. (2013) compared the time-based lateral expansion of two sizes (25 and 40) of C Point obturation points with a similar-sized gutta-percha point (control) at various distances from the point apex: 5, 10, and 15 mm under 50x magnification, using a binocular microscope. Changes in C Point dimension were significantly higher for both sizes at each tip distance after 20 min of water immersion but gutta-percha did not significantly change from the dry value during water immersion [31].

## 8. Smart Coatings for Dental Implants

Researchers at North Carolina State University have developed a “smart coating” that helps surgical implants bond more closely with bone and ward off infection. This has opened the doors to safer hip, knee, and dental implants.

When patients have hip, knee, or dental replacement surgery, they run the risk of having their bodies reject the implant. But the smart coating developed at NC State mitigates that risk by fostering bone growth into the implant. The coating creates a crystalline layer next to the implant and a mostly amorphous outer layer that touches the surrounding bone. The amorphous layer dissolves over time, releasing calcium and phosphate, which encourages bone growth.

“The bone grows into the coating as the amorphous layer dissolves, resulting in improved bonding, or osseointegration.” This bonding also makes the implant more functional, because the bonding helps ensure that the bone and the implant do a better job of sharing the load.

“We call it a smart coating because we can tailor the rate at which the amorphous layer dissolves to match the bone growth rate of each patient,” Rabiei says. This is important because people have very different rates of bone growth. For example, young people's bones tend to grow far faster than the bones of older adults.

The researchers have also incorporated silver nanoparticles throughout the coating to ward off infections. Currently, implant patients are subjected to an intense regimen of antibiotics to prevent infection immediately following surgery. However, the site of the implant will always remain vulnerable to infection. But by incorporating silver into the coating, the silver particles will act as antimicrobial agents as the amorphous layer dissolves. This not only will limit the amount of antibiotics patients will need following surgery, but also will provide protection from infection at the implant site for the life of the implant. Moreover, the silver is released more quickly right after surgery, when there is more risk of infection, due to the faster dissolution of the amorphous layer of the coating. Silver release will slow down while the patient is healing. That is another reason why the authors call it smart coating [32].

## 9. Conclusion

There is much room for the improvement and further development of materials used in dentistry. The most sophisticated class of smart materials in the foreseeable future will be that which emulate biological systems. This class of multifunctional materials will possess the capability to select and execute specific functions intelligently in order to respond to changes in the local environment. The benefit for the patient and the quality of dental therapy will undergo a significant improvement if such materials are developed and introduced.
