# Corrigendum to “Biosmart Materials: Breaking New Ground in Dentistry”

**DOI:** 10.1155/2020/7291341

**Published:** 2020-03-15

**Authors:** The Scientific World Journal

The article titled “Biosmart Materials: Breaking New Ground in Dentistry” [1] was found to contain material from previously published work. The articles are as follows:

- F. McCabe, Z. Yan, O. T. Al Naimi, G. Mahmoud, and S. L. Rolland, “Smart materials in dentistry,” Australian Dental Journal, vol. 56, no. 1, supplement, pp. 3–10, 2011. https://doi.org/10.1111/j.1834-7819.2010.01291.x. [2] (Cited as reference 23)
- K. D. Jandt and B. W. Sigusch, “Future perspectives of resin-based dental materials,” Dental Materials, vol. 25, no. 8, pp. 1001–1006, 2009. https://doi.org/10.1016/j.dental.2009.02.009. [3] (Cited as reference 11)
- A. Didato, A. A. Eid, M. D. Levin, S. Khan, F. R. Tay, and F. A. Rueggeberg, “Time-based lateral hygroscopic expansion of a water-expandable endodontic obturation point,” Journal of Dentistry, vol. 41, no. 9, pp. 796–801, 2013. https://doi.org/10.1016/j.jdent.2013.06.012. [4] (Cited as reference 31)
- J. R. Kelly and P. Benetti, “Ceramic materials in dentistry: historical evolution and current practice,” Australian Dental Journal, vol. 56, no. 1, pp. 84–96, 2011. https://doi.org/10.1111/j.1834-7819.2010.01299.x. [5] (Cited as reference 17)
- North Carolina State University, “Smart coating opens door to safer hip, knee and dental implants,” ScienceDaily. ScienceDaily, 9 February 2010. [6] (Not cited)

The article [1] includes an overlap of 425 words with J. F. McCabe et al. [2], 386 words with K. D. Jandt et al. [3], 357 words with A. Didato et al. [4], 355 words with J. R. Kelly et al. [5], and 341 words with North Carolina State University [6].

The Scientific World Journal regrets that this text reuse was not identified before publication. The authors do not agree to the publication of the corrigendum.
